# Comparative Evaluation of Non-invasive Predictors of Oesophageal Varices in Cirrhosis: Platelet Count, Spleen Diameter, and Platelet-to-Spleen Diameter Ratio

**DOI:** 10.7759/cureus.117358

**Published:** 2026-10-02

**Authors:** Basamma Dhotre, Suresh Harsoor

**Affiliations:** 1 General Medicine, Mahadevappa Rampure Medical College, Kalaburagi, IND; 2 Internal Medicine, Mahadevappa Rampure Medical College, Kalaburagi, IND

**Keywords:** liver cirrhosis, oesophageal and gastric varices, platelet count, portal hypertension, splenomegaly, ultrasonography

## Abstract

Background

Oesophageal varices are a common complication of portal hypertension in cirrhosis, and upper gastrointestinal endoscopy remains the standard method for their detection and grading. The platelet count-to-spleen diameter (PC/SD) ratio is a simple non-invasive parameter that may help identify patients at increased risk of oesophageal varices.

Methods

This prospective observational study included 80 patients with cirrhosis and portal hypertension who were evaluated at Basaveshwar Teaching and General Hospital, Kalaburagi, Karnataka, between February 20, 2024, and August 20, 2025. PC, SD measured by ultrasonography, and the PC/SD ratio were assessed. All participants underwent upper gastrointestinal endoscopy for detection and grading of oesophageal varices. The association between PC/SD ratio and variceal presence and grade was evaluated, and receiver operating characteristic (ROC) curve analysis was performed.

Results

Oesophageal varices were present in 42 (52.5%) patients, while 38 (47.5%) had no varices. Endoscopic grading showed Grade 1 varices in four (5.0%), Grade 2 in six (7.5%), and Grade 3 in 32 (40.0%) patients. The mean PC/SD ratio was significantly lower in patients with varices than in those without varices (809.51 ± 405.64 vs. 2,209.19 ± 1,479.72, p < 0.001). The mean PC/SD ratio progressively decreased with increasing variceal grade, from 2,209.19 in Grade 0 to 707.61 in Grade 3 (F = 11.887, p < 0.001). ROC analysis demonstrated an area under the curve of 0.893 (95% CI, 0.818-0.969; p < 0.001). A PC/SD ratio cut-off of <926 yielded a sensitivity of 89.5% and specificity of 69.0%.

Conclusion

The PC/SD ratio was significantly associated with the presence and severity of oesophageal varices in patients with cirrhosis and portal hypertension. It may serve as a simple adjunctive screening and risk-stratification tool to help prioritise patients for endoscopic evaluation. Larger multicentre studies are required to validate the study-specific cut-off and its broader clinical applicability.

## Introduction

Liver cirrhosis represents the advanced stage of chronic liver disease and is characterised by diffuse hepatic fibrosis, regenerative nodules, and distortion of the normal hepatic architecture. A major consequence of cirrhosis is the development of portal hypertension, resulting from increased intrahepatic vascular resistance and increased portal venous inflow. Progressive portal hypertension promotes the formation of portosystemic collateral channels, among which oesophageal varices are among the most clinically important complications because their rupture can result in life-threatening upper gastrointestinal bleeding [1,2].

Oesophageal varices are dilated submucosal veins, predominantly located in the distal oesophagus, that develop in response to portal hypertension. They occur in approximately 40-60% of patients with cirrhosis, with their prevalence increasing with advancing liver disease [3]. Variceal haemorrhage is one of the most serious complications of portal hypertension and is associated with substantial morbidity and mortality. Despite advances in the management of acute variceal bleeding and secondary prophylaxis, the first episode of variceal haemorrhage continues to be associated with a considerable risk of death [4].

Upper gastrointestinal endoscopy remains the reference standard for the detection and grading of oesophageal varices and is important for risk stratification and management planning [5]. However, routine endoscopic assessment may be challenging, particularly in resource-limited settings, because it is invasive, requires specialised equipment and trained personnel, may cause patient discomfort, and involves additional healthcare costs. These limitations have stimulated interest in simple, inexpensive, and readily available non-invasive parameters that can help identify patients at increased likelihood of having oesophageal varices and assist in prioritising those who require endoscopic evaluation [6].

Several clinical, laboratory, and radiological parameters have been investigated as non-invasive predictors of oesophageal varices. Among these, platelet count (PC) and spleen diameter (SD) are widely studied because both are closely related to the pathophysiological changes associated with portal hypertension. Thrombocytopenia in cirrhosis is commonly related to hypersplenism and splenic sequestration secondary to portal hypertension, together with reduced hepatic thrombopoietin production. Splenomegaly reflects increased portal venous pressure and splenic congestion [7]. Although PC and SD individually demonstrate associations with the presence of oesophageal varices, their diagnostic performance may be influenced by other clinical and disease-related factors.

Composite indices combining PC and SD have therefore been evaluated to improve non-invasive prediction. The PC/SD ratio integrates two readily available parameters that reflect different aspects of portal hypertension. Giannini et al. first proposed the PC/SD ratio in 2003 and demonstrated its potential value in identifying patients without oesophageal varices, suggesting that it could assist in reducing unnecessary endoscopic procedures [8].

Subsequent studies and meta-analyses have evaluated the diagnostic performance of the PC/SD ratio and have generally demonstrated useful discriminatory ability, although considerable variability has been reported across different populations and study designs [9,10]. Reported optimal cut-off values have differed according to geographic region, underlying aetiology and severity of cirrhosis, patient characteristics, disease prevalence, and methodological differences between studies. Therefore, a single PC/SD threshold may not be universally applicable across all clinical populations. In addition, most studies have primarily focused on the presence or absence of varices, while assessment of the relationship between non-invasive parameters and the endoscopic grade of oesophageal varices may provide additional clinical information relevant to risk stratification.

The potential value of non-invasive predictors is particularly relevant in resource-constrained settings where access to endoscopic services may be limited. However, such parameters should be considered as adjunctive risk-stratification tools rather than replacements for endoscopic assessment, particularly because endoscopy remains important for direct detection and grading of varices and for guiding management decisions [5,6]. Direct comparison of PC, SD, and the PC/SD ratio within the same patient population may help clarify their relative diagnostic performance and practical utility.

Therefore, the present study aimed to compare the diagnostic performance of PC, SD, and the PC/SD ratio for identifying oesophageal varices in patients with cirrhosis. In addition, the study evaluated the relationship between the PC/SD ratio and the endoscopic grade of oesophageal varices and assessed the discriminatory performance of these non-invasive parameters using receiver operating characteristic (ROC) curve analysis. The findings were intended to determine whether these routinely available parameters could serve as useful adjunctive tools for non-invasive risk stratification and prioritisation of patients for endoscopic evaluation.

## Materials and methods

Study design and setting

This prospective observational study was conducted in the Department of General Medicine at Basaveshwar Teaching and General Hospital, attached to Mahadevappa Rampure Medical College, Kalaburagi, Karnataka, India. The study was conducted over a period of 18 months, from 20 February 2024 to 20 August 2025. Patients attending the outpatient and inpatient services during the study period who fulfilled the predefined eligibility criteria were screened for participation. The study was designed to comparatively evaluate PC, SD, and the PC/SD ratio as non-invasive predictors of oesophageal varices in patients with cirrhosis and portal hypertension.

Sample size and sampling

The sample size was calculated based on the reported prevalence of oesophageal varices among patients with cirrhosis, which ranges from approximately 60% to 80%. A prevalence of 70% was considered for the calculation, with the complementary proportion of 30%. The permissible error was fixed at 15% of the assumed prevalence, corresponding to an absolute precision of approximately 10.5%. Using the formula n = Z2PQ/L2n = Z^2^PQ/L^2^, where ZZ was 1.96 for a 95% confidence level, PP was 70%, QQ was 30%, and LL was 10.5%, the calculated sample size was 73. The sample size was subsequently rounded to 80, and a total of 80 patients were included in the study. Eligible participants were recruited using simple random sampling until the required sample size was achieved.

Eligibility criteria

Patients newly diagnosed with cirrhosis and portal hypertension during the study period were eligible for inclusion. Patients aged 18 years or older and of either sex were considered eligible. Cirrhosis was diagnosed on the basis of the clinical, laboratory, and imaging findings available during routine evaluation, while portal hypertension was assessed from clinical and radiological findings, including splenomegaly, ascites, or collateral circulation.

Patients were excluded if they were haemodynamically unstable, particularly those presenting with acute variceal bleeding at admission; had a previous history of variceal bleeding, endoscopic sclerotherapy, or band ligation; had previously undergone surgery or an interventional procedure for portal hypertension, including transjugular intrahepatic portosystemic shunt or shunt surgery; were receiving pharmacological therapy for primary prophylaxis of variceal bleeding; had severe psychiatric illness; or were unwilling to participate in the study.

Clinical and laboratory assessment

All enrolled patients underwent detailed clinical evaluation, including history-taking and physical examination. Demographic characteristics, including age and sex, and the aetiology of cirrhosis were recorded using a structured study proforma. Laboratory investigations included liver function tests, complete blood count, serum albumin estimation, and prothrombin time with international normalised ratio. PCs were obtained from the complete blood count performed during the clinical evaluation. PCs were measured using an automated cell counter and were cross-checked manually when required.

Ultrasonographic assessment

Abdominal ultrasonography was performed in all participants for assessment of splenic size and measurement of SD. The maximum bipolar SD was measured in millimetres and was used for calculation of the PC/SD ratio. The mean SD in the study population was 12.795 ± 2.659 cm, equivalent to 127.95 ± 26.59 mm. Ultrasonographic assessment also categorised spleen size as normal, mild, moderate, or massive according to the assessment recorded during the study.

Calculation of the PC/SD ratio

The PC/SD ratio was calculated for each participant by dividing the PC obtained from the complete blood count by the maximum bipolar SD measured by ultrasonography in millimetres. The formula used was:

PC/SD ratio = platelet count (cells/mm³) / maximum spleen diameter (mm)

The ratio combines thrombocytopenia and splenic enlargement, two clinically relevant manifestations associated with portal hypertension.

Upper gastrointestinal endoscopy

All participants underwent upper gastrointestinal endoscopy for detection and grading of oesophageal varices. Endoscopic findings were recorded with respect to the presence or absence of oesophageal varices and their grade. For analysis, patients without oesophageal varices were classified as Grade 0, while patients with oesophageal varices were classified as Grade 1, Grade 2, or Grade 3 according to the grading recorded during endoscopic assessment.

The final study population included 38 patients without oesophageal varices, four with Grade 1 varices, six with Grade 2 varices, and 32 with Grade 3 varices. Thus, oesophageal varices were present in 42 of the 80 participants.

Study outcomes

The primary outcome of the study was the presence of oesophageal varices on upper gastrointestinal endoscopy. The secondary outcome was the relationship between the PC/SD ratio and the endoscopic grade of oesophageal varices. The diagnostic performance of PC, SD, and PC/SD ratio for identifying patients with oesophageal varices was assessed using receiver operating characteristic (ROC) curve analysis.

Statistical analysis

Data collected during the study were entered into Microsoft Excel (Microsoft Corporation, Redmond, WA, USA) and compiled into a master chart. Statistical analysis was performed using IBM SPSS Statistics for Windows, version 25.0 (IBM Corp., Armonk, NY, USA). Continuous variables were expressed as mean ± standard deviation, while categorical variables were presented as frequencies and percentages. The normality of continuous variables was assessed before application of parametric statistical tests.

Continuous variables were compared between patients with and without oesophageal varices using the independent-samples Student's t-test where appropriate. Comparisons of continuous variables across the four endoscopic variceal grades were performed using one-way analysis of variance (ANOVA). Categorical variables were analysed using the chi-square test or Fisher's exact test, as appropriate. Correlation analysis was also performed where applicable. A two-sided p-value of less than 0.05 was considered statistically significant.

Receiver operating characteristic curve analysis was performed to evaluate the diagnostic performance of PC, SD, and PC/SD ratio for prediction of oesophageal varices. The area under the ROC curve (AUC), 95% confidence interval, sensitivity, specificity, and optimal cut-off value were assessed. The optimal cut-off was determined from the ROC analysis based on the balance between sensitivity and specificity.

The relationship between PC/SD ratio and endoscopic variceal grade was additionally assessed. This analysis was performed because the study evaluated not only the presence or absence of oesophageal varices but also their endoscopic severity.

Ethical considerations

The study was conducted after obtaining approval from the Institutional Ethics Committee of Mahadevappa Rampure Medical College, Kalaburagi (EC No. 2024167). Written informed consent was obtained from all participants in their native language before enrolment. Participation was voluntary, and confidentiality of participant identity and clinical information was maintained throughout the study. The study procedures were conducted in accordance with the approved study protocol and applicable ethical principles.

## Results

Study population and baseline characteristics

A total of 80 patients with cirrhosis and portal hypertension were included in the study. The age distribution is presented in Table 1. After verification of the study records, no participant was younger than 18 years; the youngest age category was therefore corrected to 18-29 years. Five patients (6.25%) were aged 18-29 years, 24 (30.0%) were aged 30-39 years, 18 (22.5%) were aged 40-49 years, 21 (26.25%) were aged 50-59 years, and 12 (15.0%) were aged 60 years or older. The study population was predominantly male, with 73 males (91.3%) and seven females (8.8%) (Table 1).

**Table 1:** Baseline demographic characteristics of the study population (n = 80) Data are presented as frequencies (n) and percentages (%). Percentages may not total exactly 100% because of rounding.

Variable	Category	Frequency, n	Percentage, %
Age (years)	18–29	5	6.25
	30–39	24	30.00
	40–49	18	22.50
	50–59	21	26.25
	≥60	12	15.00
Sex	Male	73	91.3
	Female	7	8.8

The distribution of the major demographic characteristics is also illustrated in Figure 1.

**Figure 1:**
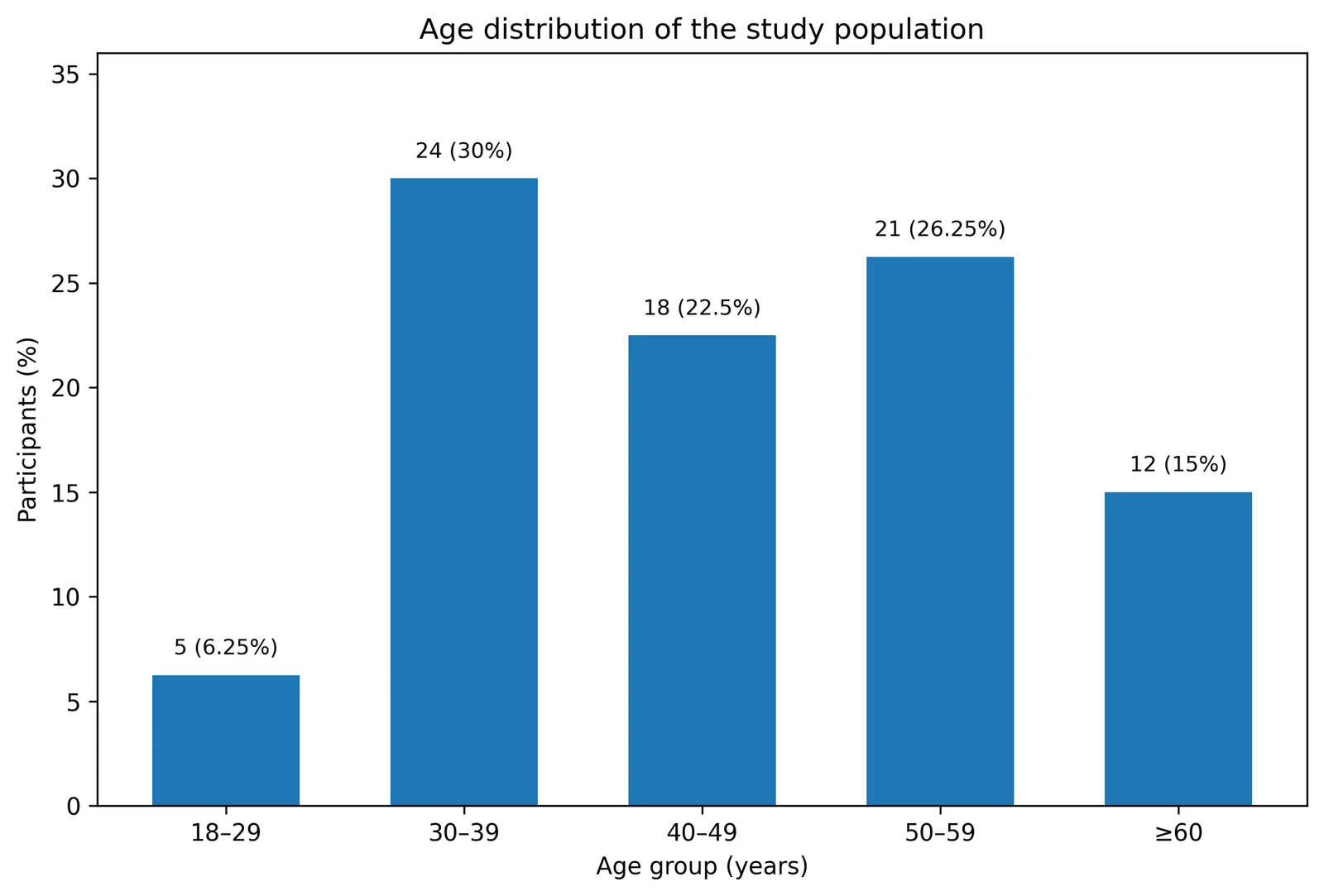
Age distribution of the study population Bars represent the percentage of participants in each age category; numbers above the bars indicate the frequency and percentage. The age category was corrected to 18–29 years after verification of the original study records.

Clinical characteristics and aetiology of cirrhosis

On clinical examination, splenomegaly was present in 41 patients (51.2%), whereas 39 patients (48.8%) did not have clinically detectable splenomegaly. Ultrasonography demonstrated splenomegaly in 46 patients (57.5%), while 34 patients (42.5%) had a normal spleen size. Based on ultrasonographic assessment, 17 patients (21.3%) had mild splenomegaly, 23 (28.7%) had moderate splenomegaly, and six (7.5%) had massive splenomegaly (Table 2).

**Table 2:** Clinical and ultrasonographic characteristics of the study population (n = 80) Grade 0 indicates the absence of oesophageal varices. Grades 1, 2, and 3 represent the endoscopic grades recorded in the study. Values above the bars indicate frequency and percentage.

Variable	Category	Frequency, n	Percentage, %
Clinical splenomegaly	Present	41	51.2
	Absent	39	48.8
Ultrasonographic splenomegaly	Present	46	57.5
	Absent	34	42.5
Ultrasonographic spleen size	Normal	34	42.5
	Mild splenomegaly	17	21.3
	Moderate splenomegaly	23	28.7
	Massive splenomegaly	6	7.5
Aetiology	Alcohol-related	31	38.8
	Viral hepatitis	25	31.3
	NASH/metabolic	11	13.8
	Other/cryptogenic	9	11.3
	Autoimmune/cholestatic	4	5.0
Ascites	None	19	23.8
	Mild	25	31.3
	Moderate to severe	36	45.0
Icterus	Present	52	65.0
	Absent	28	35.0

Alcohol-related liver disease was the most common aetiology, accounting for 31 patients (38.8%), followed by viral hepatitis in 25 (31.3%), NASH/metabolic causes in 11 (13.8%), other or cryptogenic causes in nine (11.3%), and autoimmune or cholestatic liver disease in four (5.0%). Ascites was present in 61 patients (76.3%), including moderate-to-severe ascites in 36 (45.0%) and mild ascites in 25 (31.3%). Nineteen patients (23.8%) had no ascites. Icterus was present in 52 patients (65.0%), while 28 (35.0%) had no icterus.

The clinical and ultrasonographic distribution of splenomegaly is shown in Figure 2.

**Figure 2:**
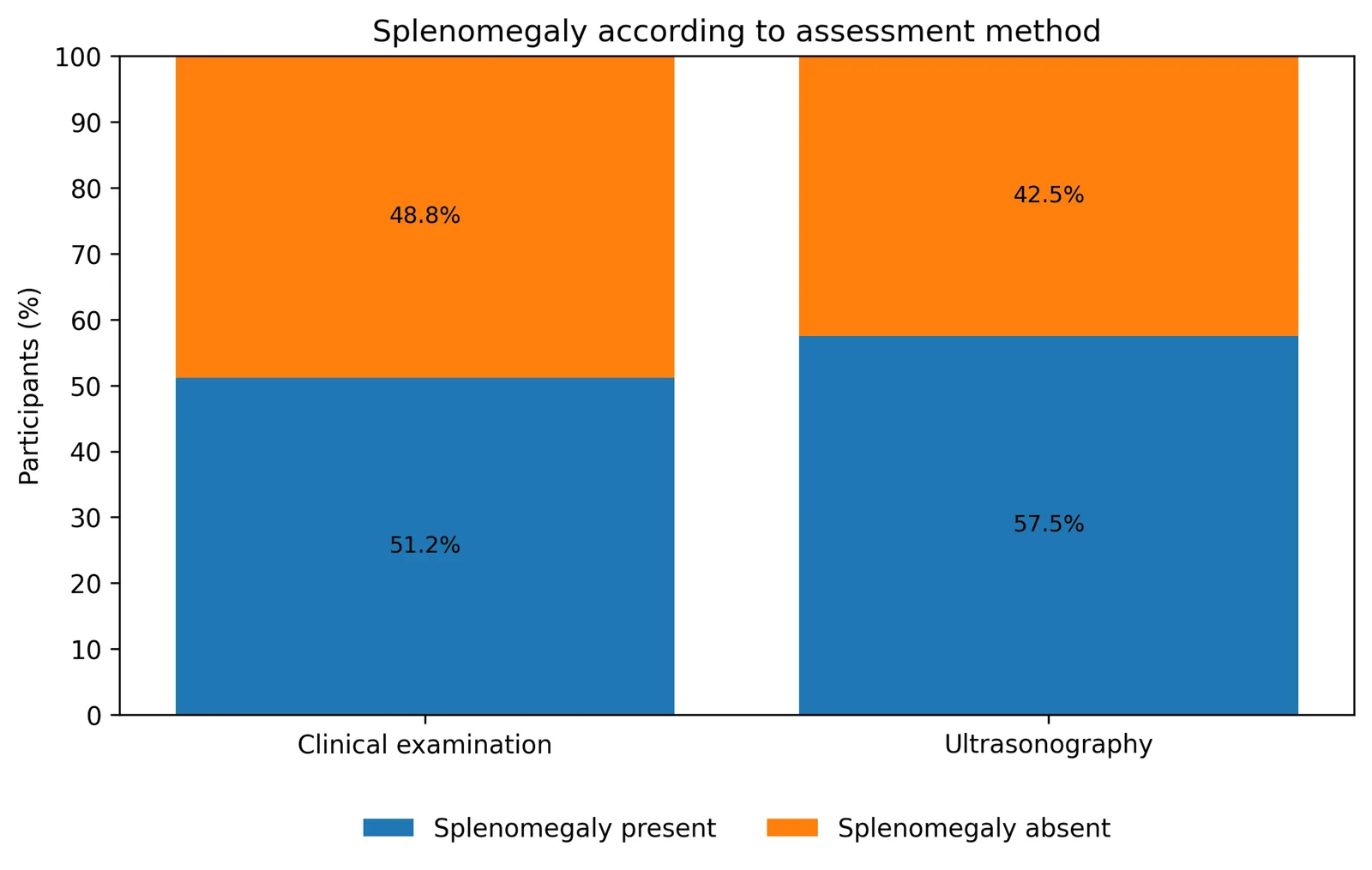
Splenomegaly according to assessment method Bars represent the proportion of participants with and without splenomegaly according to clinical examination and ultrasonography.

Laboratory and liver disease severity parameters

The mean haemoglobin concentration was 8.817 ± 2.140 g/dL, while the mean total leukocyte count was 29,737.86 ± 16,951.29 cells/mm³. The mean PSR for the overall study population was 1474.35 ± 1267.13. The mean total bilirubin was 5.551 ± 5.410 mg/dL, direct bilirubin was 2.4065 ± 3.1067 mg/dL, indirect bilirubin was 1.780 ± 1.397 mg/dL, aspartate aminotransferase (AST) was 65.85 ± 48.16 IU/L, alanine aminotransferase (ALT) was 40.404 ± 45.17 IU/L, and serum albumin was 2.694 ± 2.161 g/dL. The mean international normalised ratio (INR) was 2.547 ± 0.763.

The mean SD measured by ultrasonography was 12.795 ± 2.659 cm, corresponding to 127.95 ± 26.59 mm. The laboratory and ultrasonographic parameters are summarised in Table 3.

**Table 3:** Laboratory and ultrasonographic parameters of the study population Data are presented as mean ± standard deviation. AST, aspartate aminotransferase; ALT, alanine aminotransferase; INR, international normalised ratio; PSR, platelet count-to-spleen diameter ratio

Parameter	Mean ± SD	Unit
Haemoglobin	8.817 ± 2.140	g/dL
Total leukocyte count	29,737.86 ± 16,951.29	cells/mm³
Total bilirubin	5.551 ± 5.410	mg/dL
Direct bilirubin	2.4065 ± 3.1067	mg/dL
Indirect bilirubin	1.780 ± 1.397	mg/dL
AST	65.85 ± 48.16	IU/L
ALT	40.404 ± 45.17	IU/L
Serum albumin	2.694 ± 2.161	g/dL
INR	2.547 ± 0.763	Ratio
Spleen diameter	12.795 ± 2.659	cm
Spleen diameter	127.95 ± 26.59	mm
PSR	1474.35 ± 1267.13	—

Hepatic encephalopathy was absent in 45 patients (56.3%), whereas 14 patients (17.5%) had Grade I-II and 21 patients (26.3%) had Grade III-IV encephalopathy. According to the Child-Pugh classification, six patients (7.5%) were classified as Child-Pugh class A, 18 (22.5%) as class B, and 56 (70.0%) as class C. The mean Child-Pugh score was 11.20 ± 3.037 (Table 4).

**Table 4:** Hepatic encephalopathy and Child-Pugh classification Data are presented as frequency (n) and percentage (%) except for the Child-Pugh score, which is presented as mean ± standard deviation.

Variable	Category	Frequency, n	Percentage, %
Hepatic encephalopathy	None	45	56.3
	Grades I–II	14	17.5
	Grades III–IV	21	26.3
Child-Pugh class	A	6	7.5
	B	18	22.5
	C	56	70.0
Child-Pugh score	Mean ± SD	11.20 ± 3.037	—

Endoscopic findings and grading of oesophageal varices

Upper gastrointestinal endoscopy demonstrated oesophageal varices in 42 of the 80 patients (52.5%), while 38 patients (47.5%) had no oesophageal varices. Among the complete study population, 38 patients (47.5%) had Grade 0 findings, four (5.0%) had Grade 1 varices, six (7.5%) had Grade 2 varices, and 32 (40.0%) had Grade 3 varices (Table 5).

**Table 5:** Endoscopic distribution and grade of oesophageal varices (n = 80) Grade 0 represents absence of oesophageal varices. Grades 1–3 represent the endoscopic grades recorded in the study. Data are presented as frequency (n) and percentage (%).

Variceal grade	Frequency, n	Percentage, %
Grade 0: No varices	38	47.5
Grade 1	4	5.0
Grade 2	6	7.5
Grade 3	32	40.0
Total	80	100

Thus, 42 patients (52.5%) had endoscopically detected oesophageal varices, with Grade 3 varices accounting for the largest proportion of patients with varices (32/42, 76.2%). The overall distribution of oesophageal varices and their grades is shown in Figure 3.

**Figure 3:**
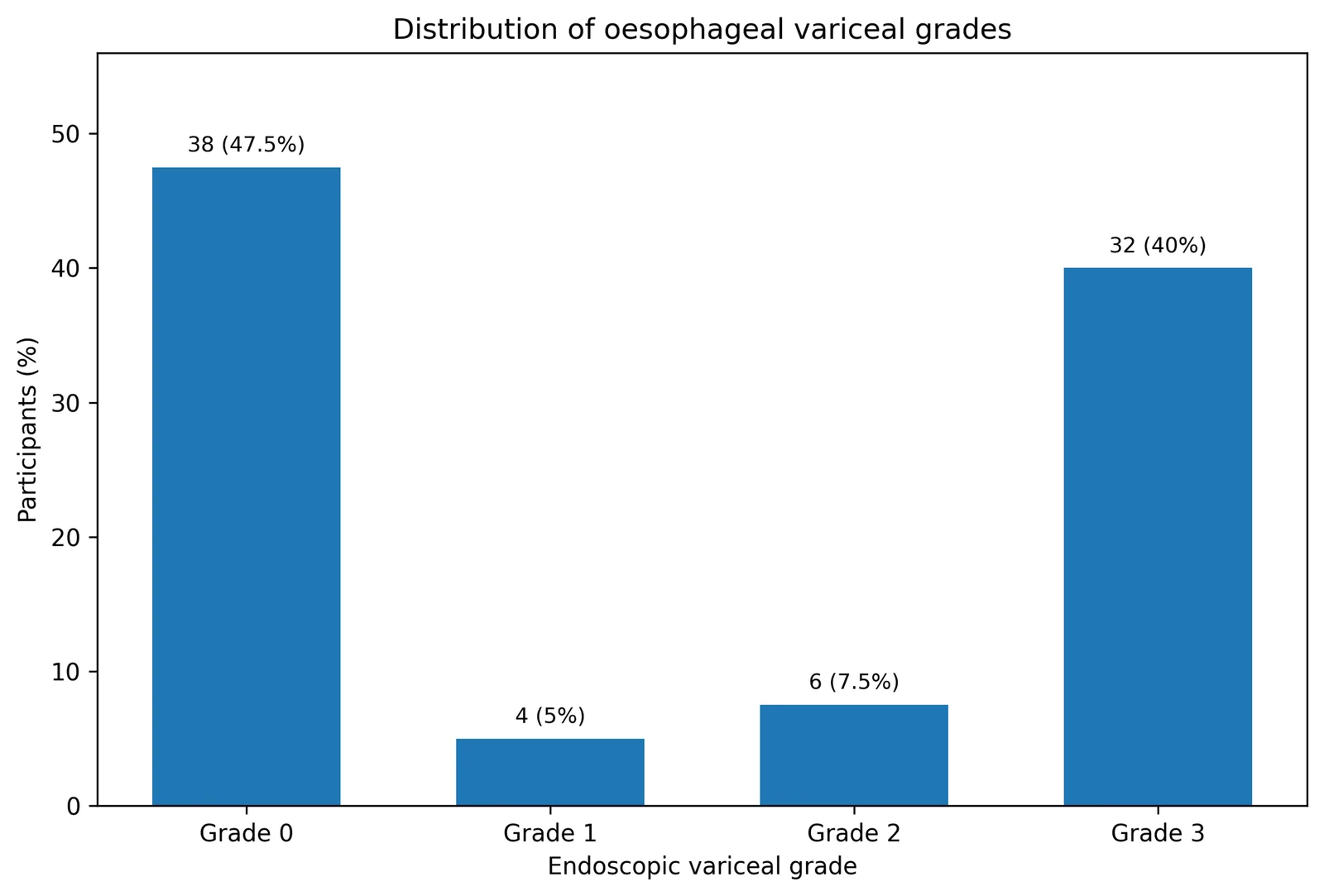
Distribution of oesophageal variceal grades Grade 0 indicates absence of oesophageal varices. Grades 1, 2, and 3 represent the endoscopic grades recorded in the study. Values above the bars indicate frequency and percentage.

Association between PSR and presence of oesophageal varices

The mean PSR differed significantly between patients with and without oesophageal varices. Patients without varices had a mean PSR of 2209.19 ± 1479.72, whereas patients with varices had a substantially lower mean PSR of 809.51 ± 405.64. The difference was statistically significant on Student's t-test (p < 0.001) (Table 6).

**Table 6:** Comparison of PSR according to the presence of oesophageal varices Data are presented as mean ± standard deviation. The comparison was performed using Student's t-test. A p-value <0.05 was considered statistically significant. PSR, platelet count-to-spleen diameter ratio

Endoscopic finding	Mean PSR ± SD	p-value
No oesophageal varices	2209.19 ± 1479.72	<0.001
Oesophageal varices present	809.51 ± 405.64	
Overall	1474.35 ± 1267.13	

The comparison of PSR between patients with and without oesophageal varices is illustrated in Figure 4.

**Figure 4:**
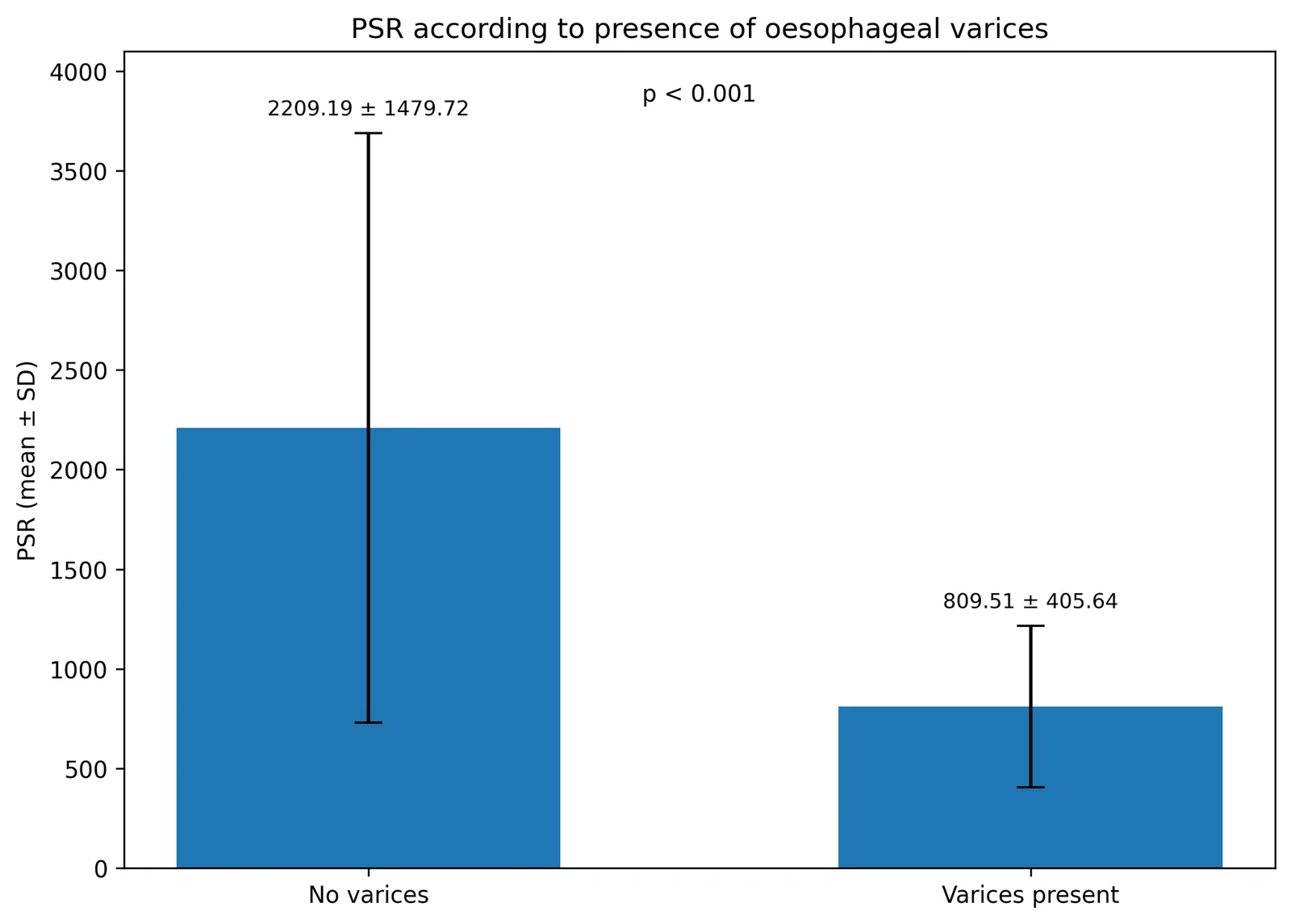
PSR according to the presence of oesophageal varices Bars represent mean PSR values and error bars represent standard deviation. PSR, platelet count-to-spleen diameter ratio. The difference between groups was statistically significant (p < 0.001).

Association between PSR and grade of oesophageal varices

A progressive reduction in mean PSR was observed with increasing endoscopic grade of oesophageal varices. The mean PSR was 2209.19 ± 1479.72 in patients without varices (Grade 0), 1203.93 ± 240.52 in patients with Grade 1 varices, 1089.99 ± 526.02 in patients with Grade 2 varices, and 707.61 ± 348.40 in patients with Grade 3 varices (Table 7). One-way ANOVA demonstrated a statistically significant difference in PSR across the four groups (F = 11.887, p < 0.001).

**Table 7:** Association between PSR and endoscopic grade of oesophageal varices Data are presented as mean ± standard deviation. Grade 0 represents patients without oesophageal varices. Differences across the four groups were assessed using one-way analysis of variance (ANOVA). PSR, platelet count-to-spleen diameter ratio. F = 11.887; p < 0.001.

Variceal grade	Mean PSR	SD
Grade 0	2209.19	1479.72
Grade 1	1203.93	240.52
Grade 2	1089.99	526.02
Grade 3	707.61	348.40
Overall	1474.36	1267.13

The inverse relationship between PSR and variceal grade is illustrated in Figure 5.

**Figure 5:**
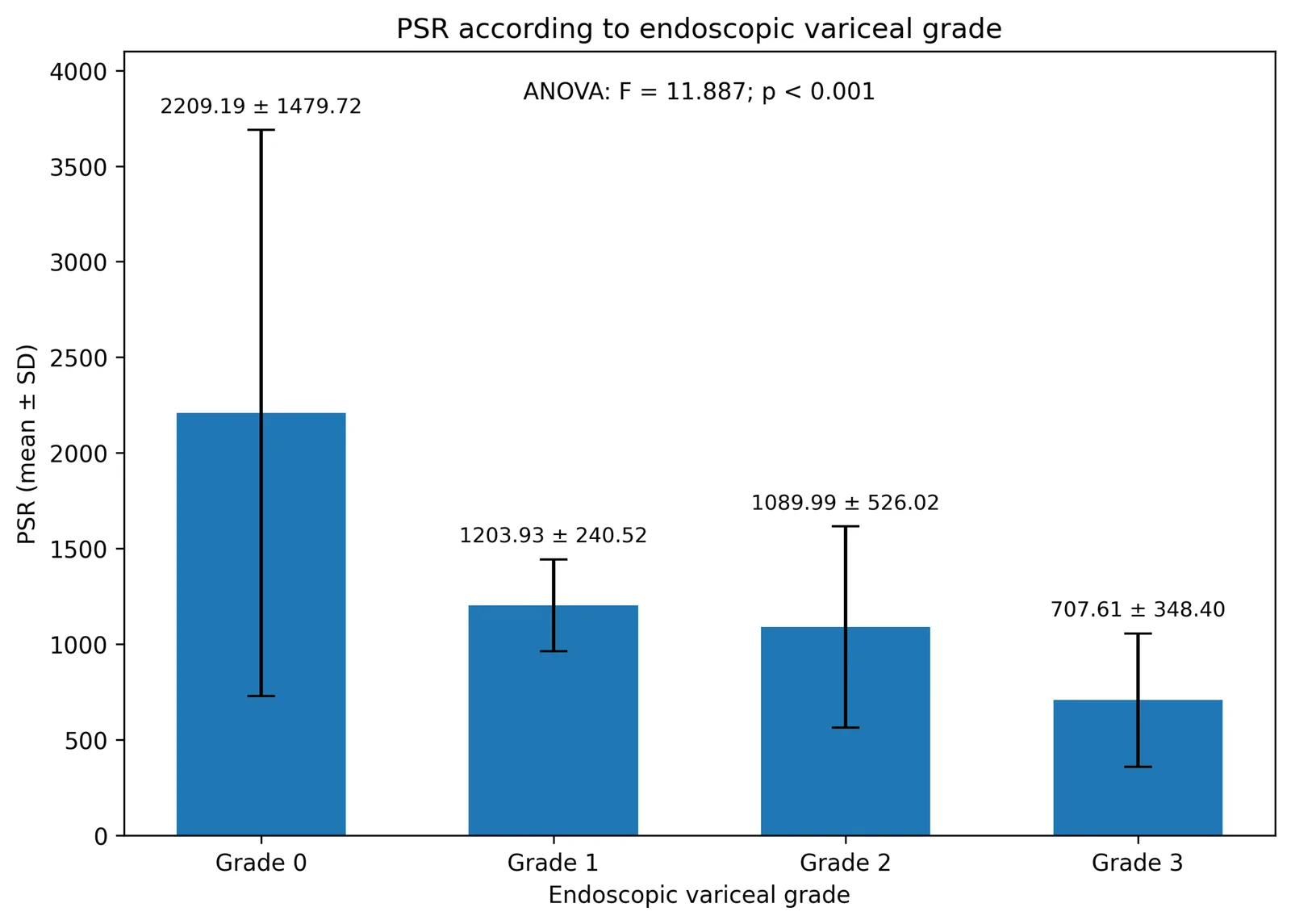
PSR according to endoscopic variceal grade Bars represent mean PSR values and error bars represent standard deviation. PSR progressively decreased across increasing grades of oesophageal varices. One-way ANOVA demonstrated a statistically significant difference across groups (F = 11.887, p < 0.001). PSR, platelet count-to-spleen diameter ratio

Diagnostic performance of PSR

Receiver operating characteristic curve analysis demonstrated good discriminatory performance of PSR for predicting the presence of oesophageal varices. The area under the ROC curve was 0.893, with a standard error of 0.038 and a 95% confidence interval of 0.818-0.969 (p < 0.001) (Table 8).

**Table 8:** Receiver operating characteristic analysis of PSR for prediction of oesophageal varices The reported cut-off was <926. Sensitivity and specificity were calculated for the prediction of the presence of oesophageal varices. AUC, area under the receiver operating characteristic curve; CI, confidence interval; PSR, platelet count-to-spleen diameter ratio

Parameter	Value
Area under the curve	0.893
Standard error	0.038
95% confidence interval	0.818–0.969
p-value	<0.001
Optimal PSR cut-off	<926
Sensitivity	89.5%
Specificity	69.0%

A PSR cut-off value of <926 identified patients with oesophageal varices with a sensitivity of 89.5% and specificity of 69.0%. The ROC curve for PSR is presented in Figure 6.

**Figure 6:**
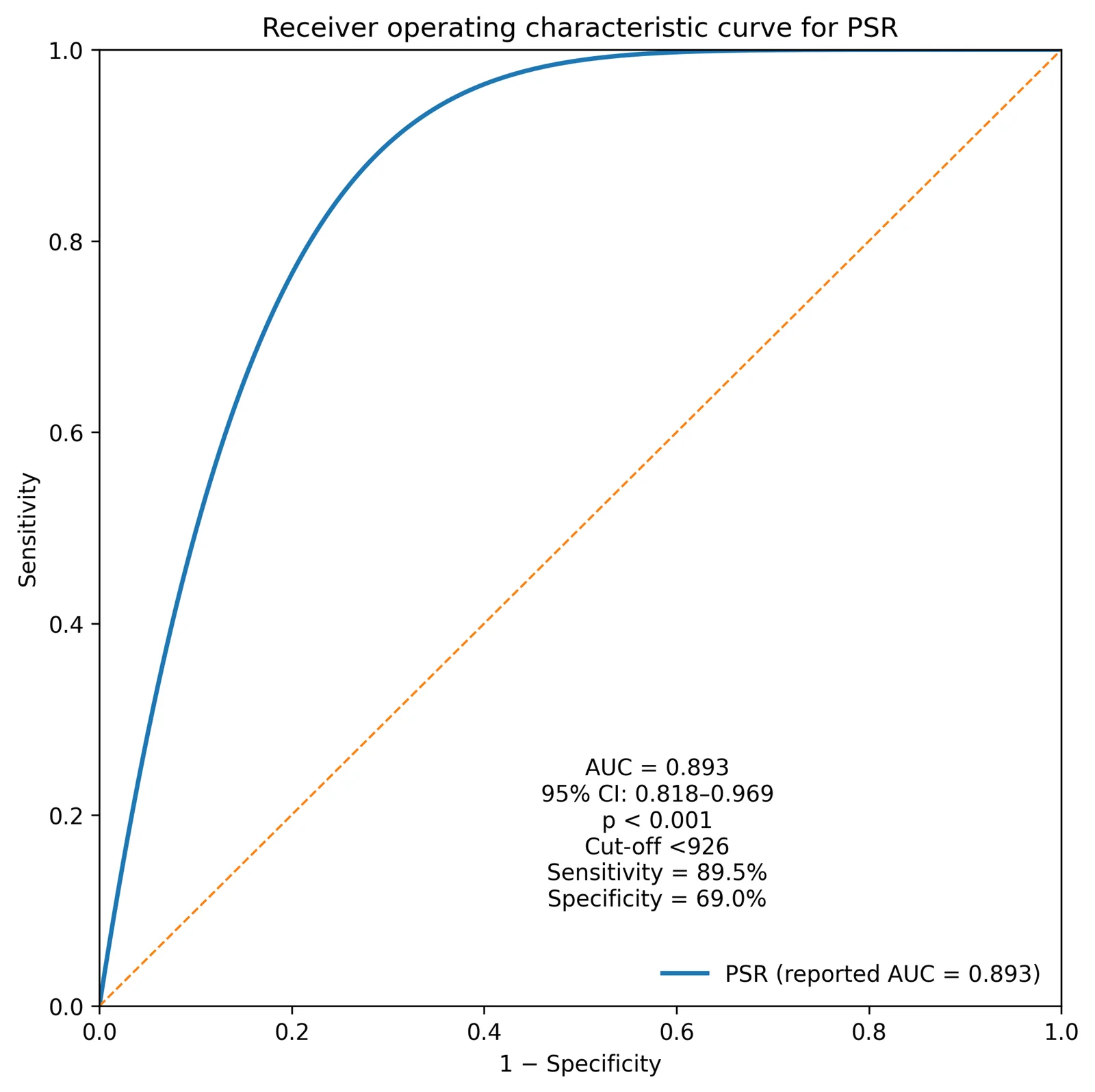
Receiver operating characteristic curve for PSR in predicting oesophageal varices The AUC was 0.893 (95% CI, 0.818–0.969; p < 0.001), with a reported PSR cut-off of <926, sensitivity of 89.5%, and specificity of 69.0%. PSR, platelet count-to-spleen diameter ratio; AUC, area under the curve; CI, confidence interval

The ROC analysis demonstrated that lower PSR values were associated with the presence of oesophageal varices, with an AUC of 0.893 and statistically significant discrimination between patients with and without varices. The observed progressive decline in PSR across increasing grades of oesophageal varices further demonstrated an inverse association between PSR and endoscopic variceal severity.

## Discussion

The present prospective observational study evaluated PC, SD, and the PC/SD ratio as non-invasive predictors of oesophageal varices in patients with cirrhosis and portal hypertension. Oesophageal varices were identified in 42 of 80 patients (52.5%). The PC/SD ratio was significantly lower in patients with varices than in those without varices and also demonstrated a progressive decline with increasing endoscopic variceal grade. These findings indicate an inverse relationship between the PC/SD ratio and the presence and severity of oesophageal varices.

The pathophysiological basis of the PC/SD ratio relates to the effects of portal hypertension on the spleen and PC. Splenic congestion and enlargement occur with increasing portal pressure, while hypersplenism and reduced hepatic thrombopoietin production contribute to thrombocytopenia. Giannini et al. proposed the PC-to-SD ratio as a non-invasive parameter for predicting oesophageal varices and reported a cut-off value of 909 [5]. This provided the basis for subsequent studies evaluating the reproducibility and diagnostic utility of the ratio in different populations. The relationship between splenomegaly and thrombocytopenia in cirrhosis has also been described, supporting the rationale for combining these two parameters into a composite index [6].

Several subsequent studies have demonstrated an association between lower PC/SD values and the presence of oesophageal varices. Sarangapani et al. reported the usefulness of the ratio for predicting large oesophageal varices in patients with chronic liver disease [7]. A subsequent multicentre validation study further evaluated the ratio across different centres and supported its potential as a non-invasive predictor of oesophageal varices [8]. The findings of the present study are consistent with these observations, as patients with oesophageal varices had substantially lower PC/SD values than those without varices.

The diagnostic performance of the PC/SD ratio has also been evaluated in systematic reviews and meta-analyses. Chawla et al. reported useful pooled sensitivity and specificity of the ratio for predicting oesophageal varices, supporting its potential role as a non-invasive screening parameter [9]. Similarly, Baig et al. reported favourable diagnostic performance of the PC/SD ratio across studies evaluating patients with cirrhosis [10]. Nevertheless, differences between study populations, underlying aetiology, severity of liver disease, measurement methods, and diagnostic thresholds contribute to heterogeneity in reported performance.

In the present study, the mean PC/SD ratio was 2209.19 ± 1479.72 in patients without varices compared with 809.51 ± 405.64 in patients with varices (p < 0.001). Importantly, the ratio also demonstrated a progressive reduction with increasing endoscopic grade, from 2209.19 ± 1479.72 in patients without varices to 1203.93 ± 240.52 in Grade 1, 1089.99 ± 526.02 in Grade 2, and 707.61 ± 348.40 in Grade 3 varices. This association was statistically significant on one-way analysis of variance (F = 11.887, p < 0.001). These findings suggest that the PC/SD ratio may provide information not only about the presence of varices but also about their endoscopic severity. Mangone et al. demonstrated the usefulness of the ratio in patients with compensated cirrhosis, although the diagnostic performance may differ according to the characteristics of the population studied [11]. Barrera et al. similarly evaluated the PC/SD ratio for predicting high-risk oesophageal varices and reported its potential clinical value in identifying patients with more clinically significant variceal disease [12].

ROC analysis in the present study demonstrated an area under the curve of 0.893 (95% CI, 0.818-0.969; p < 0.001). A PC/SD ratio cut-off of <926 yielded a sensitivity of 89.5% and specificity of 69%. The cut-off observed in the present study is broadly comparable with values reported in previous studies, including the original threshold of 909 proposed by Giannini et al. [5]. However, optimal PC/SD thresholds have varied between studies, and differences may arise from geographical variation, underlying aetiology of cirrhosis, disease severity, patient characteristics, and measurement techniques [7,11,12]. Therefore, the cut-off of <926 should be considered a study-specific threshold and should not be assumed to have universal applicability without external validation.

Non-invasive assessment of portal hypertension has expanded beyond conventional platelet and ultrasonographic parameters. Ultrasound elastography and measurements of liver and spleen stiffness have been investigated as additional approaches for assessing portal hypertension and predicting oesophageal varices [13]. These techniques may provide complementary information regarding portal hypertension and liver disease severity. However, their availability may be limited in some clinical settings because they require specialised equipment and technical expertise.

Current management strategies continue to recognise endoscopic assessment as an important component of evaluation for oesophageal varices in appropriate patients with cirrhosis [14]. More recent evidence has further emphasised the potential role of liver stiffness, spleen stiffness, and combined non-invasive models in identifying patients at risk of clinically significant portal hypertension [15]. Similarly, clinical guidelines for variceal haemorrhage continue to support appropriate endoscopic evaluation and management when indicated [16]. Therefore, the PC/SD ratio should be regarded as an adjunctive screening and risk-stratification parameter rather than a replacement for endoscopy.

An important consideration in interpreting the PC/SD ratio is that PC is influenced by factors other than portal hypertension. Alcohol-related bone marrow suppression, viral infections, nutritional deficiencies, medications, chronic inflammation, and reduced hepatic thrombopoietin production may contribute to thrombocytopenia and consequently influence the calculated ratio [17]. SD may also be affected by individual anatomical variation and differences in ultrasonographic measurement. These factors may contribute to variability in PC/SD values and should be considered when applying the ratio to individual patients.

Previous reviews of non-invasive screening have similarly concluded that although several parameters can help identify patients at increased risk of oesophageal varices, no single non-invasive marker can consistently replace endoscopic assessment across all patient populations [18]. In this context, the PC/SD ratio may have practical value because it can be calculated from routinely available PCs and spleen measurements without requiring additional specialised investigations. It may therefore help identify patients who warrant prioritised endoscopic evaluation, particularly in settings where endoscopic resources are limited.

The present study has several strengths. Its prospective design allowed systematic assessment of patients undergoing evaluation for cirrhosis and portal hypertension. PC, SD, and the PC/SD ratio were evaluated within the same study population, allowing direct comparison of these parameters. In addition, all participants underwent upper gastrointestinal endoscopy, and the analysis included endoscopic variceal grading rather than limiting the outcome to the presence or absence of varices. The significant inverse association between PC/SD ratio and variceal grade adds clinical information to the assessment of the ratio.

Several limitations should be considered. The study included only 80 patients and was conducted at a single centre, which limits generalisability and external validation. Potential factors influencing PC were not independently controlled. Variability in ultrasonographic spleen measurements may also influence the calculated ratio. Furthermore, the number of patients in the lower variceal-grade categories was relatively small. The study did not evaluate other contemporary non-invasive modalities, such as transient elastography or spleen stiffness measurements. Finally, although the PC/SD ratio demonstrated the highest observed AUC among the evaluated parameters, a formal statistical comparison between ROC curves, such as the DeLong test, was not performed. Therefore, the higher AUC should be interpreted as a descriptive finding and not as definitive statistical evidence of superiority over the individual parameters.

Overall, the findings support the PC/SD ratio as a simple and accessible adjunctive parameter associated with both the presence and severity of oesophageal varices in cirrhosis. The study-specific cut-off should, however, be interpreted cautiously. Larger multicentre studies involving diverse patient populations, standardised measurement methods, formal comparison of diagnostic models, and external validation are required before the PC/SD ratio can be considered for broader clinical application.

## Conclusions

The PC/SD ratio was significantly associated with the presence and severity of oesophageal varices in patients with cirrhosis and portal hypertension. It may serve as a simple, accessible adjunctive screening and risk-stratification tool to help prioritise patients for endoscopic evaluation. However, it should not replace endoscopy, and further multicentre studies are required to validate its clinical applicability.
